# Supplementary material for: Fast-growing species shape the evolution of reef corals
Source: Nat Commun. 2022 May 3;13:2426. doi: 10.1038/s41467-022-30234-6 (PMC9065008; doi:10.1038/s41467-022-30234-6)
Supplement: Supplementary file 1 — Supplementary Information [file 41467_2022_30234_MOESM1_ESM.pdf]

Supplementary Information for:

Fast-growing species shape the evolution of reef corals

Nature Communications

Alexandre C. Siqueira[1,2]*; Wolfgang Kiessling[3]; David R. Bellwood[1,2]

[1] Research Hub for Coral Reef Ecosystem Functions, College of Science and Engineering, James Cook University, Townsville, QLD 4811, Australia.

[2] ARC Centre of Excellence for Coral Reef Studies, James Cook University, Townsville, QLD 4811, Australia.

[3] GeoZentrum Nordbayern, Friedrich-Alexander University Erlangen - Nürnberg (FAU), Erlangen 91054, Germany.

* Author for Correspondence: Alexandre C. Siqueira, alexandre.siqueira@my.jcu.edu.au

Supplementary figures

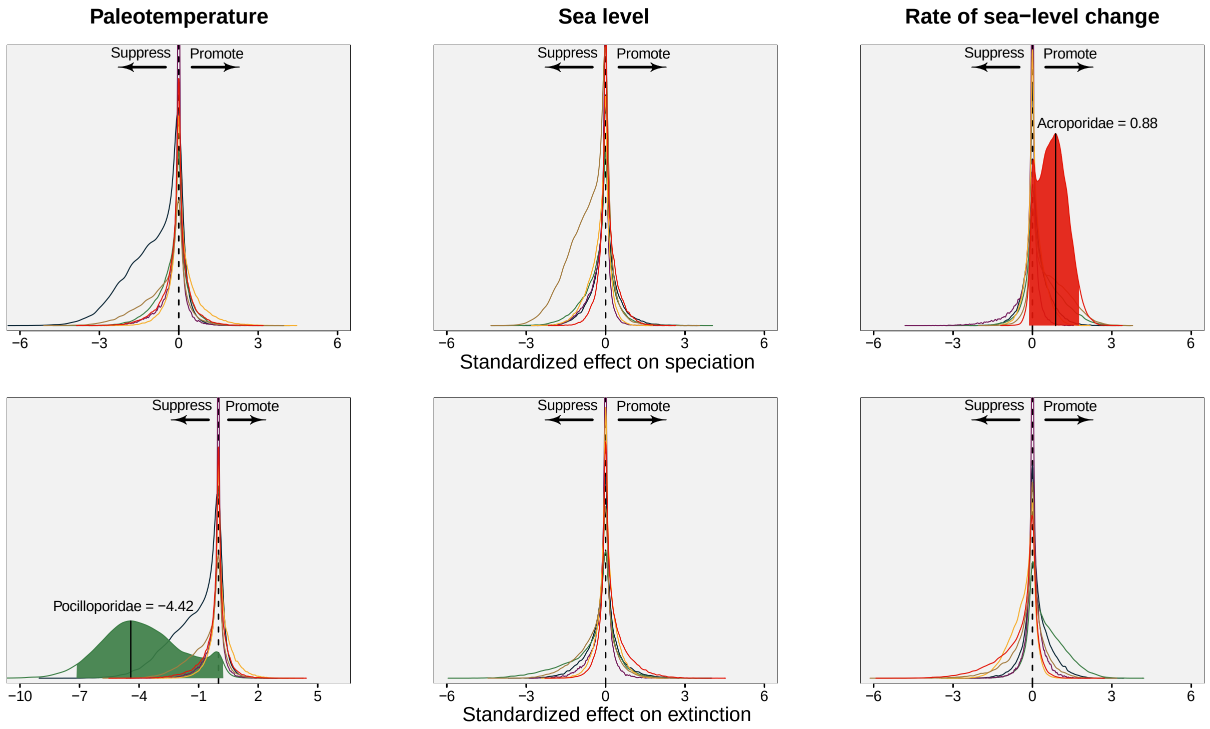

Supplementary Figure 1 | Effects of environmental variables in evolutionary rates of reef coral families. Posterior distribution of effects of paleotemperature (left), sea level (centre) and rate of sea-level change (right) in speciation (top) and extinction (bottom) rates of each of the focal scleractinian coral families. Effects were estimated through the Multivariate Birth-Death model (see Methods). Filled densities represent effects that differ from zero, from which we show the modal value (black line) along with the family name. Source data are provided as a Source Data file.

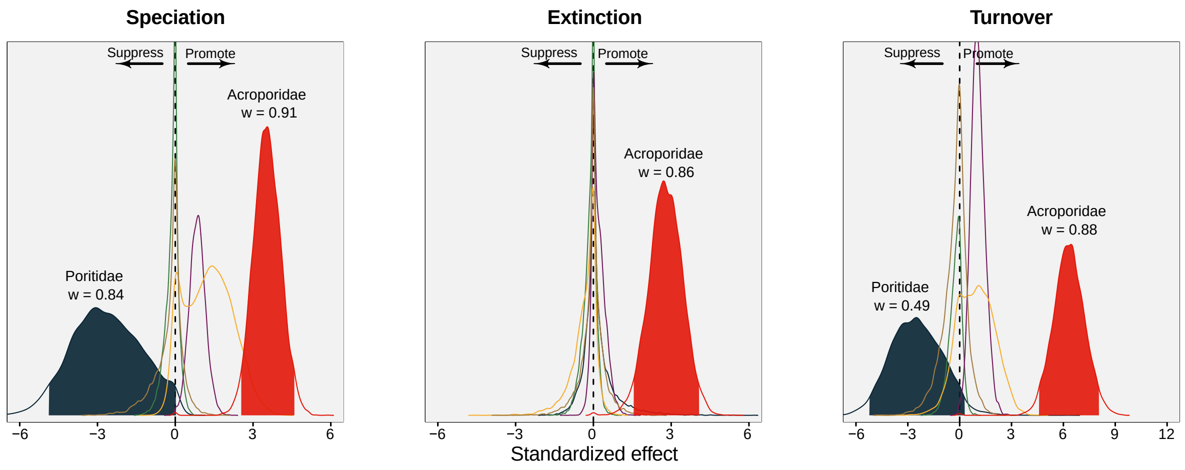

Supplementary Figure 2 | Diversity-dependent effects in overall reef coral evolutionary rates. Posterior distribution of effects on overall speciation (left), extinction (centre) and lineage turnover (right; effect on speciation plus effect on extinction). Effects were estimated through the Multivariate Birth-Death model by selecting only sites in which the Acroporidae co-occur with the other coral families (see Methods). Filled densities represent effects estimated with a strong signal ($w > 0.7$). The median shrinkage weights (w) are also shown for these families. Source data are provided as a Source Data file.

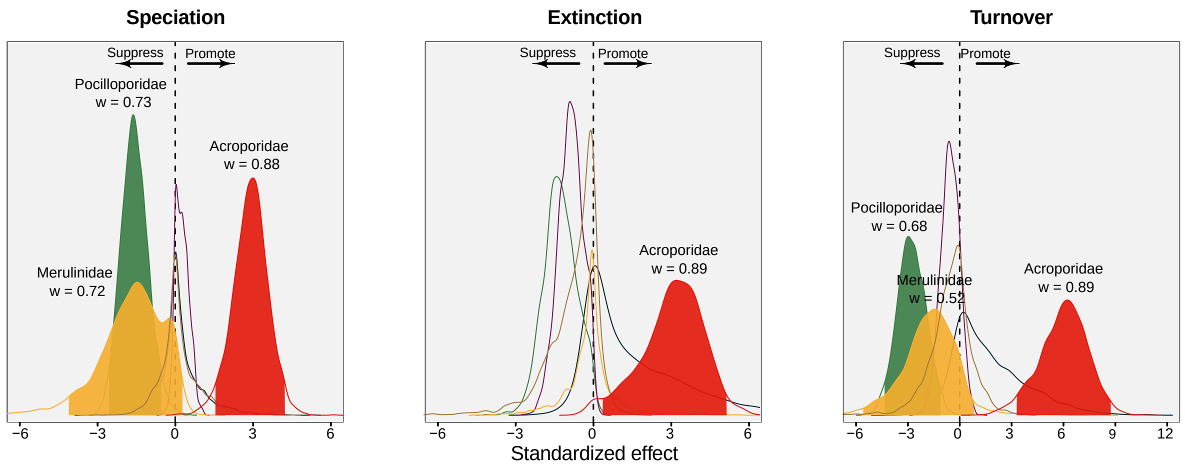

Supplementary Figure 3 | Diversity-dependent effects in overall reef coral evolutionary rates. Posterior distribution of effects on overall speciation (left), extinction (centre) and lineage turnover (right; effect on speciation plus effect on extinction). Effects were estimated through the Multivariate Birth-Death model by selecting Indo-Pacific sites only (see Methods). Filled densities represent effects estimated with a strong signal (w > 0.7). The median shrinkage weights (w) are also shown for these families. Source data are provided as a Source Data file.

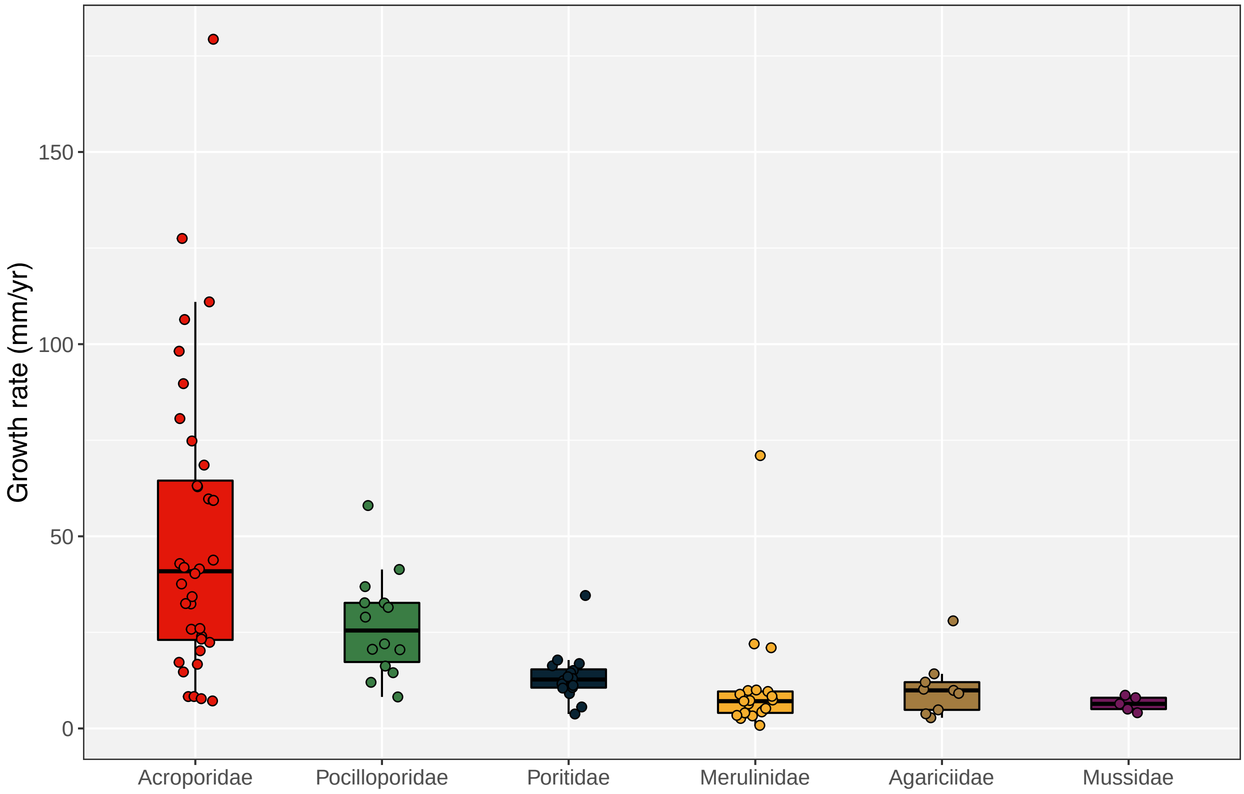

Supplementary Figure 4 | Growth rate per focal reef coral family. Distribution of growth rates, measured as linear extension rates (mm * year^{-1}), for each of the focal reef coral families. Each point represents the mean value of individual species with available growth rate data (*Acroporidae* n = 36*;* *Pocilloporidae* n = 14; *Poritidae* n = 16; *Merulinidae* n = 21; *Agariciidae* n = 9; *Mussidae* n = 5). Boxes represent the interquartile range and the black line represents the median values. Whiskers represent the range from the lowest to the largest values within 1.5 times the interquartile range. The dataset was sourced from the Coral Trait Database (https://coraltraits.org; accessed on 9 March 2022). A Generalised Linear Model (GLM) fit to these data shows that the average growth in the Acroporidae is significantly faster than the other families.

Source data are provided as a Source Data file.

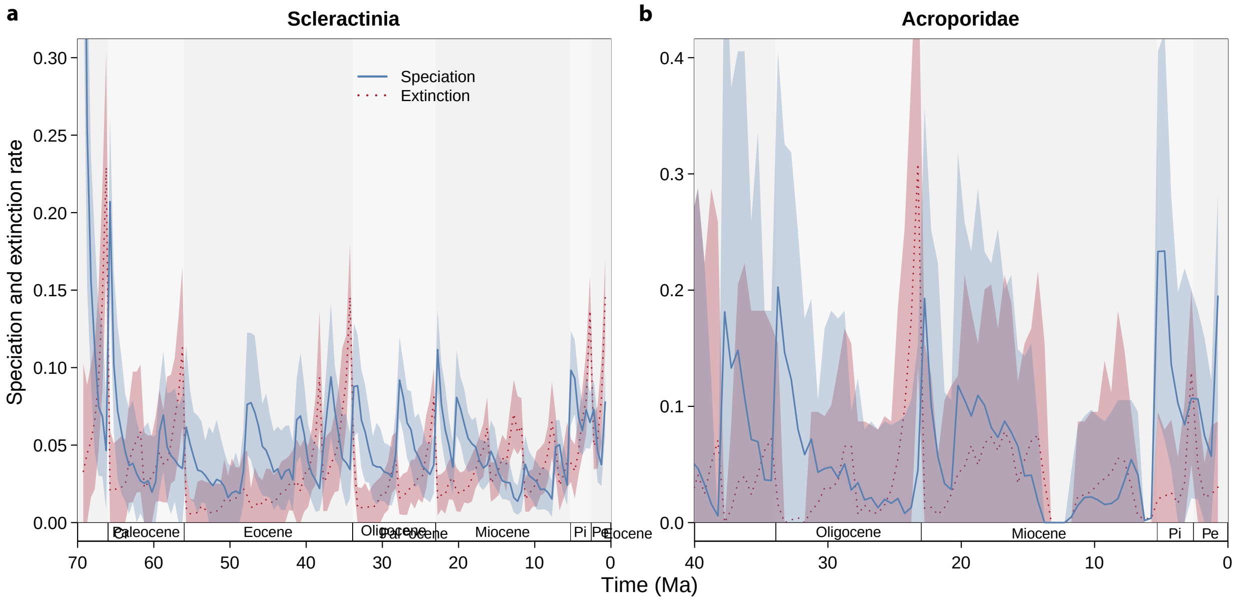

Supplementary Figure 5 | Evolutionary rates through time in Scleractinia and Acroporidae. Rates of speciation (blue) and extinction (red dashed) estimated for fossil lineages of Scleractinia (**a**; n = 4,235) and Acroporidae (**b**; n = 165) using the divDyn framework (see Methods). The analyses incorporate all the fossils recorded in the respective groups, although here we only show the last 70 million years. Solid and dashed lines represent mean rates, while coloured shadings represent 95% confidence intervals from the age resampling routine. Pi – Pliocene; Pe – Pleistocene. Source data are provided as a Source Data file.

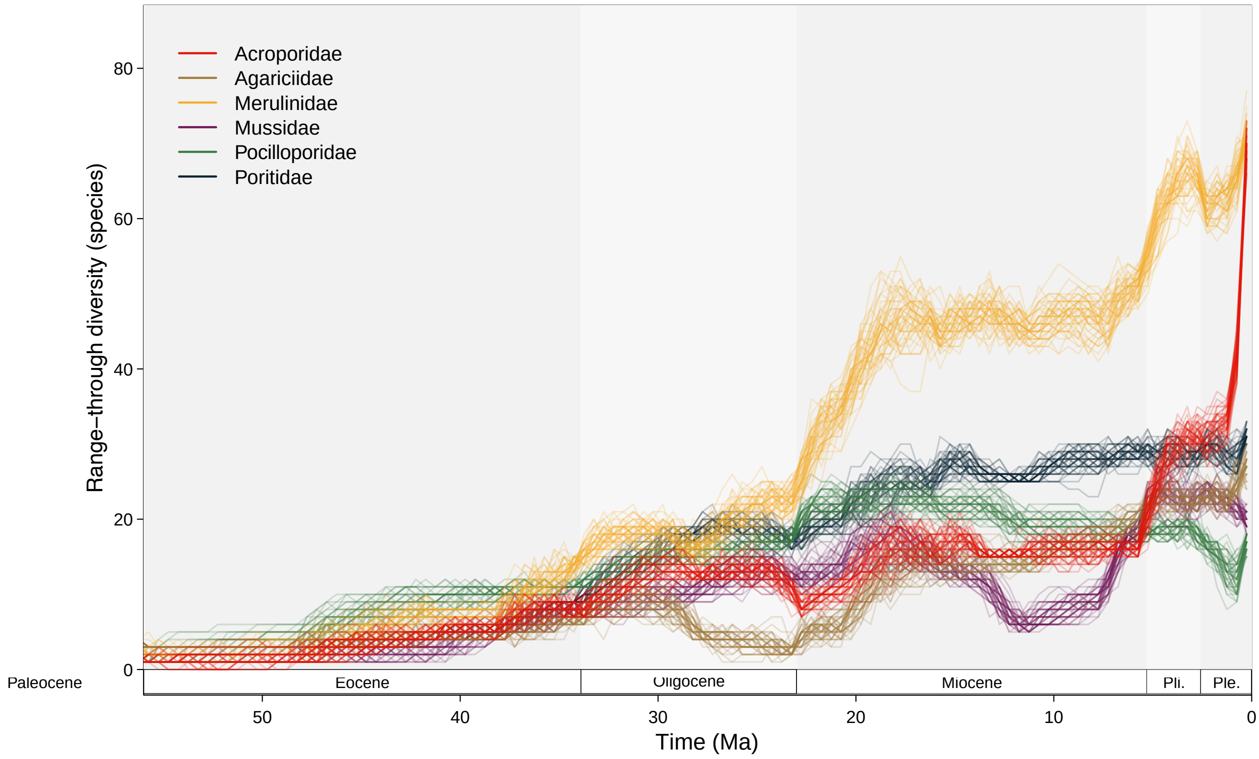

Supplementary Figure 6 | Fossil lineages through time in six extant reef coral families. Reconstruction of fossil trajectories using the Range-through diversity metric. Models were run independently in each family and were replicated fifty times (individual lines) to incorporate uncertainty around the age of the fossil occurrences (see Methods). Source data are provided as a Source Data file.

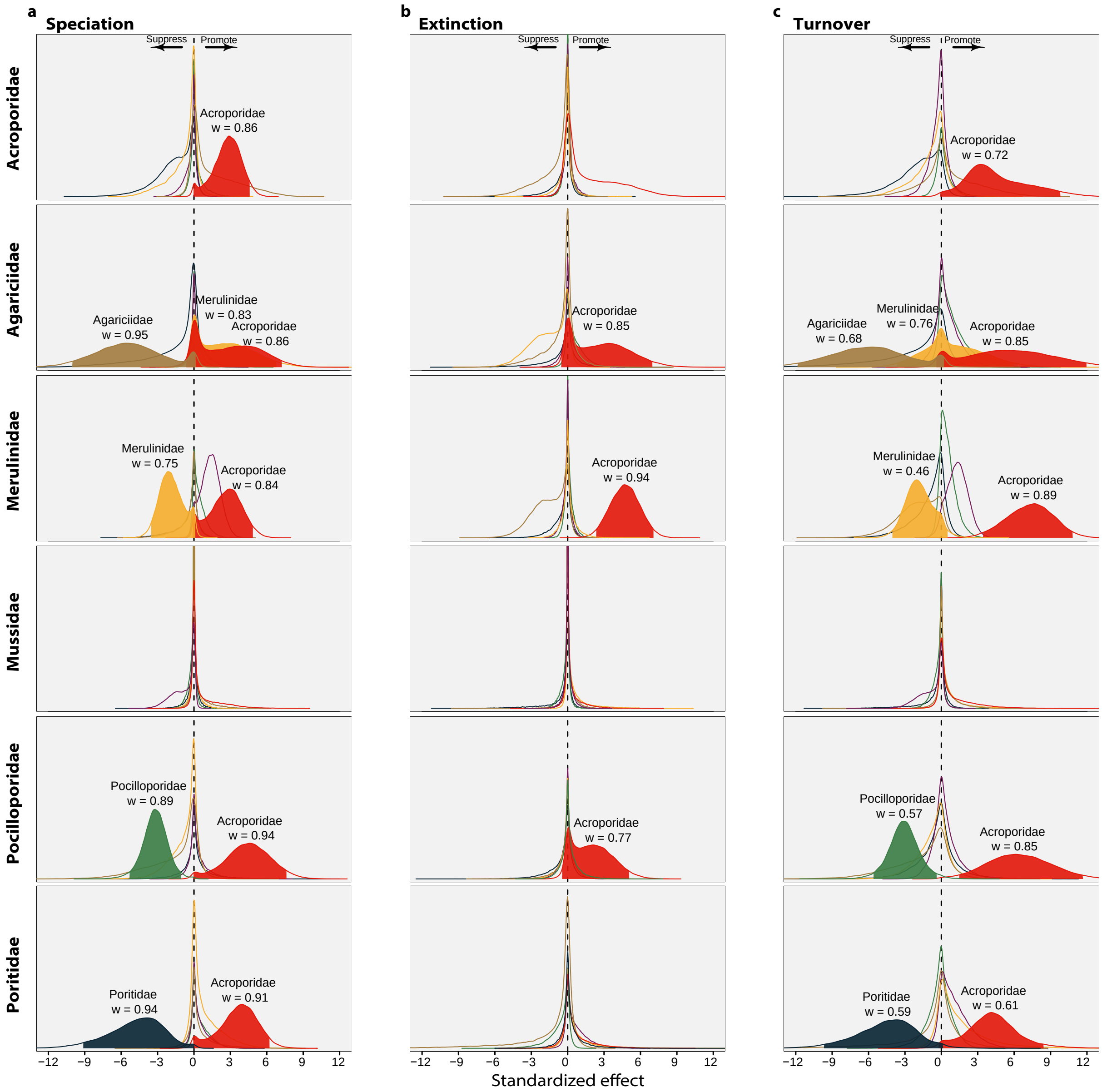

Supplementary Figure 7 | Cross-family effects in evolutionary rates. Posterior distribution of cross-family effects on speciation (left), extinction (centre) and lineage turnover (right; effect on speciation plus effect on extinction). Effects were estimated through a Multivariate Birth-Death model by reef coral family (rows), in which the diversity trajectories of all other families (including self-diversity dependency) were included as predictors (see Methods). Filled densities represent effects estimated with a strong signal (w > 0.7). The median shrinkage weights (w) are also shown for these families.

Supplementary Tables

Supplementary Table 1 | Median shrinkage weights (w) and correlation parameters (Cor) estimated across speciation and extinction effects per reef coral family for the main Multivariate Birth-Death model, along with respective lower and upper 95% confidence intervals (CI).

	Median w	CI lower w	CI upper w	Median Cor	CI lower Cor	CI upper Cor
**Acroporidae**	0.9415371	0.7691933	0.9955636	4.613238	3.455761	5.660047
**Agariciidae**	0.5330895	0.0046238	0.9753363	-0.697983	-3.890375	0.433034
**Merulinidae**	0.2428835	0.0018159	0.9289252	0.187103	-0.390664	2.016042
**Mussidae**	0.1343647	0.0011483	0.8477131	0.115061	-0.234718	0.762498
**Pocilloporidae**	0.3159372	0.0040913	0.9199201	0.265592	-0.589375	1.362203
**Poritidae**	0.7946212	0.0234826	0.9872496	-2.274612	-4.489239	0.026455

Supplementary Table 2 | Preservation model comparison across fossil data subsets. We compared three models of fossil preservation (see Methods): the homogeneous Poisson process (HPP); the nonhomogeneous Poisson process (NHPP); and the time-variable Poisson process (TPP). The AICc values were substantially lower for the TPP model (ΔAICc > 50), supporting a time-variable model of preservation across all data subsets.

	AICc HPP	AICc NHPP	AICc TPP
**Subset 1**	8980.58	9016.96	8868.57
**Subset 2**	11843.00	12759.01	10972.07
**Subset 3**	10590.42	10687.91	10462.51
**Subset 4**	12101.81	13339.23	11241.23
**Subset 5**	9748.24	9981.70	9636.30
**Subset 6**	9042.08	9546.83	8719.23
**Subset 7**	10383.16	25146.20	9414.09
**Subset 8**	9507.14	9573.76	9267.56
**Subset 9**	10256.95	11964.42	9588.08
**Subset 10**	9451.23	9510.39	9329.91
**Subset 11**	10286.71	10914.34	9759.80

Supplementary Table 3 | Preservation model comparison across reef coral families. We compared three models of fossil preservation (see Methods): the homogeneous Poisson process (HPP); the nonhomogeneous Poisson process (NHPP); and the time-variable Poisson process (TPP). The AICc values were substantially lower for the TPP model (ΔAICc > 50), supporting a time-variable model of preservation across all families.

	AICc HPP	AICc NHPP	AICc TPP
**Acroporidae**	1800.52	1812.08	1743.97
**Agariciidae**	937.54	1093.75	838.32
**Merulinidae**	6542.24	9732.34	5016.40
**Mussidae**	1570.31	1785.02	1460.51
**Pocilloporidae**	2553.96	2932.29	2204.20
**Poritidae**	4070.52	4524.34	3732.03
