# Supplementary material for: Fast-growing species shape the evolution of reef corals
Source: Nat Commun. 2022 May 3;13:2426. doi: 10.1038/s41467-022-30234-6 (PMC9065008; doi:10.1038/s41467-022-30234-6)
Supplement: Supplementary file 3 — Reporting Summary [file 41467_2022_30234_MOESM3_ESM.pdf]

nature portfolio

Corresponding author(s): Alexandre C. Siqueira

Last updated by author(s): 04/04/2022

Reporting Summary

Nature Portfolio wishes to improve the reproducibility of the work that we publish. This form provides structure for consistency and transparency in reporting. For further information on Nature Portfolio policies, see our Editorial Policies and the Editorial Policy Checklist.

Statistics

For all statistical analyses, confirm that the following items are present in the figure legend, table legend, main text, or Methods section.

n/a	Confirmed	
☐	☒	The exact sample size (*n*) for each experimental group/condition, given as a discrete number and unit of measurement
☒	☐	A statement on whether measurements were taken from distinct samples or whether the same sample was measured repeatedly
☐	☒	The statistical test(s) used AND whether they are one- or two-sided<br>*Only common tests should be described solely by name; describe more complex techniques in the Methods section.*
☐	☒	A description of all covariates tested
☐	☒	A description of any assumptions or corrections, such as tests of normality and adjustment for multiple comparisons
☐	☒	A full description of the statistical parameters including central tendency (e.g. means) or other basic estimates (e.g. regression coefficient) AND variation (e.g. standard deviation) or associated estimates of uncertainty (e.g. confidence intervals)
☒	☐	For null hypothesis testing, the test statistic (e.g. *F*, *t*, *r*) with confidence intervals, effect sizes, degrees of freedom and *P* value noted<br>*Give P values as exact values whenever suitable.*
☐	☒	For Bayesian analysis, information on the choice of priors and Markov chain Monte Carlo settings
☐	☒	For hierarchical and complex designs, identification of the appropriate level for tests and full reporting of outcomes
☒	☐	Estimates of effect sizes (e.g. Cohen's *d*, Pearson's *r*), indicating how they were calculated

Our web collection on statistics for biologists contains articles on many of the points above.

Software and code

Policy information about availability of computer code

Data collection	The software R (version 4.0.3) was used to process data and scripts are provided in the Zenodo repository (see Data).
Data analysis	The PyRate program (version 3.0; available at github.com/dsilvestro/PyRate) was used for data analysis and is written in Python (version 3.). The software Tracer (version 1.7.1) is available at github.com/beast-dev/tracer/releases/tag/v1.7.1. The software R (version 4.0.3) was also used to perform data analyses. The R packages used were: tidyverse (version 1.3.1); scales (version 1.1.1); igraph (version 1.2.6); HDInterval (version 0.2.2); and shape (version 1.4.6).

For manuscripts utilizing custom algorithms or software that are central to the research but not yet described in published literature, software must be made available to editors and reviewers. We strongly encourage code deposition in a community repository (e.g. GitHub). See the Nature Portfolio guidelines for submitting code & software for further information.

Data

Policy information about availability of data

All manuscripts must include a data availability statement. This statement should provide the following information, where applicable:

- Accession codes, unique identifiers, or web links for publicly available datasets
- A description of any restrictions on data availability
- For clinical datasets or third party data, please ensure that the statement adheres to our policy

All data was collected from the publicly available Paleobiology Database (PBDB – paleobiodb.org; accessed on 3 August 2021). The datasets generated and/or analysed during the current study are available through the Zenodo repository: https://doi.org/10.5281/zenodo.6413373.

Field-specific reporting

Please select the one below that is the best fit for your research. If you are not sure, read the appropriate sections before making your selection.

☐ Life sciences ☐ Behavioural & social sciences ☒ Ecological, evolutionary & environmental sciences

For a reference copy of the document with all sections, see nature.com/documents/nr-reporting-summary-flat.pdf

Ecological, evolutionary & environmental sciences study design

All studies must disclose on these points even when the disclosure is negative.

Study description	We assessed the diversification dynamics of scleractinian reef coral fossils at the species level to: (a) describe the major temporal changes in evolutionary rates that determined the formation of present-day species richness patterns among scleractinian coral families; (b) explore fossil diversity trajectories of major extant reef-building coral families; and (c) estimate the potential effects of environmental and biological drivers on speciation and extinction rates across reef coral families.
Research sample	The research sample was composed of all described fossil species within the order Scleractinia, along with their respective temporal occurrence data. The rationale for our sample choice is that we were interested in exploring diversification rates across all scleractinian fossils through time. This data is publicly available through the Paleobiology Database (see Data collection), the most comprehensive repository for palaeontological data in reef corals to-date.
Sampling strategy	Sample size was determined by availability of palaeontological occurrence data for reef-building corals. We used all available data in our study, with the exception of taxa with uncertain taxonomic assignments (see Data exclusions).
Data collection	All data was collected from the publicly available Paleobiology Database (PBDB – paleobiodb.org; accessed on 3 August 2021). This is a comprehensive repository that collates paleontological data for multiple taxonomic groups. In the case of scleractinian corals, most data in PBDB was collated and curated by one of the authors (Wolfgang Kiessling) based on the relevant literature.
Timing and spatial scale	The scope of this study is global. Since all data used was publicly available, there were no time constraints for data collection.
Data exclusions	To minimize identification issues, we excluded taxa with uncertain generic and species assignments (i.e., classified as aff. and cf.) and only selected species that had accepted names.
Reproducibility	Since our study is not an experiment, our results can not be replicated. However, we do provide all the relevant data and code to reproduce the results using the same framework that we utilized.
Randomization	This is not relevant to our study, because we did not perform an experiment that required group allocation. Organisms analyzed herein were chosen based on habitat and taxonomic affinities (Reef-building corals).
Blinding	This is not relevant to our study, because we did not perform an experiment that required blinding and we analysed the entire scleractinian fossil dataset.

Did the study involve field work? ☐ Yes ☒ No

Reporting for specific materials, systems and methods

We require information from authors about some types of materials, experimental systems and methods used in many studies. Here, indicate whether each material, system or method listed is relevant to your study. If you are not sure if a list item applies to your research, read the appropriate section before selecting a response.

Materials & experimental systems

n/a	Involved in the study	
☒	☐	Antibodies
☒	☐	Eukaryotic cell lines
☐	☒	Palaeontology and archaeology
☒	☐	Animals and other organisms
☒	☐	Human research participants
☒	☐	Clinical data
☒	☐	Dual use research of concern

Methods

n/a	Involved in the study	
☒	☐	ChIP-seq
☒	☐	Flow cytometry
☒	☐	MRI-based neuroimaging

Palaeontology and Archaeology

Specimen provenance	We did not collect new specimens, only used fossil data that was publicly available at the Paleobiology Database (see Data collection).
Specimen deposition	We did not deposit new specimens, only used fossil data that was publicly available (see Data collection).
Dating methods	We did not perform dating, all occurrence dates were publicly available (see Data collection).

☒ Tick this box to confirm that the raw and calibrated dates are available in the paper or in Supplementary Information.

Ethics oversight	Since the data used herein was publicly available, there was no need for Ethics oversight.

Note that full information on the approval of the study protocol must also be provided in the manuscript.
